# Cancer

**Published:** 1891-09

**Authors:** 


### CANCER.

An able medical writer says that cancer is often caused by mental strain and worry resulting from extraordinary reverses of fortune, and that novel theory is well borne out by the circumstances of the death of Frank Leslie and General Grant.

In speaking of the matter, Dr. Hodgman, one of the oldest and most successful physicians of the city, said: The instances mentioned are, indeed, singularly striking in their similarity. I am well acquainted with the circumstances surrounding Mr. Leslie's sickness and death, having attended him and family, and I have carefully studied the cases of General Grant and John Roach. I would be scarcely willing to go to the length of saying that the cancer in each of these cases was the direct result of mental strain and worry, but I have no doubt that the business reverses of these three men accelerated, if they did not superinduce, the fatal malady from which each suffered. When one's mind becomes troubled the body must become more or less debilitated, and when the body becomes debilitated any latent disease will assert itself. Yet I must believe that the germ of the disease must have been present in each case, awaiting only an opportunity for development and growth. The opportunity was offered alike to Leslie, Roach and Grant. Their minds became worried, and their bodies became weakened and the cancers appeared. Had not these reverses come, with the consequent mental trouble, those three men might have lived for years without developing the fatal disease.—*Ex.*

